# Influence of Prosthesis Position on Pacing Burden and Long-Term Survival in TAVI Patients with New Pacemaker

**DOI:** 10.3390/jcm14041265

**Published:** 2025-02-14

**Authors:** Ramona Schmitt, Yannick Salz-Sculean, Klaus Kaier, Constantin von zur Mühlen, Johannes Brado, Manuel Hein, Martin Soschynski, Christopher L. Schlett, Franz-Josef Neumann, Dirk Westermann, Philipp Ruile, Philipp Breitbart

**Affiliations:** 1 Department of Cardiology and Angiology, Medical Center-University of Freiburg, Faculty of Medicine, University of Freiburg, 79189 Bad Krozingen, Germany; 2 Institute of Medical Biometry and Statistics, Medical Center-University of Freiburg, Faculty of Medicine, University of Freiburg, 79106 Freiburg, Germany; 3 Department of Diagnostic and Interventional Radiology, Medical Center-University of Freiburg, Faculty of Medicine, University of Freiburg, 79106 Freiburg, Germany

**Keywords:** computed tomography angiography, conduction disturbances, fusion imaging, TAVI

## Abstract

**Objectives:** The aim of this study was to evaluate the influence of three-dimensional transcatheter heart valve (THV) position on mid-term pacing burdens and to examine the effect of stimulation rates on survival after transcatheter aortic valve implantation (TAVI). **Methods:** In patients receiving a new PPM before discharge after TAVI, we analyzed the final three-dimensional THV position in 107 patients (78 with Sapien S3 THV and 29 with Evolut R THV) using fusion imaging from pre- and post-TAVI computed tomography angiography. The PPM stimulation rates were examined pre-discharge and after 3 months. Patient survival was tracked over a period of 5 years. **Results:** A high pacing burden (stimulation rate of >20%) was observed in 69% of patients before discharge and in 67% after three months. One-third of the patients with an initial low pacing burden developed a high pacing burden after 3 months. The implantation depth did not differ between patients with or without a high pacing burden at discharge (*p* = 0.550) or after 3 months (*p* = 0.901). The three-dimensional THV position showed no influence on the pacing burden pre-discharge (*p* = 0.550) and after 3 months (0.901). There were no significant differences in survival, regardless of the pacing burden pre-discharge and after 3 months (log-rank *p* = 0.676). **Conclusions:** Three-dimensional THV position did not predict a high pacing burden after TAVI. Pacing burden was not associated with survival. One-third of patients experienced an increase to a high pacing burden within 3 months, suggesting potential long-term conduction system damage after TAVI.

## 1. Introduction

Transcatheter aortic valve implantation (TAVI) has become an established treatment for patients with severe aortic stenosis [1]. A common complication after TAVI is the development of new conduction disturbances, often leading to the need for permanent pacemaker (PPM) implantation [2]. High right ventricular pacing burden (PPM stimulation rate of >20%) is generally associated with poor long-term outcomes, e.g., due to the development of pacemaker-induced cardiomyopathy [3]. The authors of prior studies have suggested a strong correlation between the final prosthesis position, the extent of calcification in the device landing zone, and the occurrence of new conduction disturbances requiring PPM implantation after TAVI [4]. Thus far, data on predictors of PPM pacing burden after TAVI and its impact on long-term outcomes in these patients are scarce.

Thus, the primary aim of this study was to evaluate the impact of three-dimensional transcatheter heart valve (THV) position and device landing zone characteristics on the PPM pacing rate in patients requiring a new PPM as a complication of TAVI, both before discharge and after 3 months. The secondary objective was to assess the influence of PPM pacing burdens on the long-term outcomes of these patients.

## 2. Materials and Methods

Candidates for inclusion in this retrospective study were all patients who underwent implantation of a new PPM after TAVI and had evaluable computed tomography angiography (CTA) images of the aortic valve region before and after the procedure. Post-TAVI-CTA was performed routinely at our institution until 2022 in accordance with the guidelines available at the time of thoracic aortic stent implantation to identify possible complications, such as valve thrombosis or aortic injury [5]. Reasons for not performing post-TAVI-CTA, such as renal insufficiency, have been described previously by our group [6]. The implanted THV type was the self-expanding Evolut R (Medtronic, Minneapolis, MN, USA) and the balloon-expandable Sapien S3 (Edwards Lifesciences Corp., Irvine, CA, USA). Patients without available PPM data at pre-discharge were excluded from the analysis. Furthermore, valve-in-valve procedures were excluded, as fusion imaging (described below) is not feasible in these patients.

In all patients, eligibility for TAVI and procedural characteristics were determined in consensus by the multidisciplinary heart team at our institution. THV implantation was performed via transfemoral, transapical, or trans-subclavian access. A new conduction disturbance was defined as the new onset of or progress in a pre-existing atrioventricular block or bundle branch block. The decision for permanent pacemaker implantation was made by an experienced electrophysiologist in accordance with our institutional guidelines and guidelines of the European Society of Cardiology at the time of the TAVI procedure. Depending on the type of conduction disturbance, either a single-chamber or dual-chamber pacemaker was implanted. Pacemaker controls were conducted before discharge and after 3 months. We defined a high pacing burden as a >20% PPM stimulation rate, as recommended by the current guidelines of the European Society of Cardiology [3]. Patients were monitored using a standardized follow-up protocol including annual phone calls for up to 8 years to determine adverse events such as death, myocardial infarction, stroke, bleeding, and hospitalizations.

All clinical data and follow-up information were obtained from our institutional database and supplemented by contacting general practitioners when necessary. All patients gave their written informed consent for the anonymized use of clinical, procedural, and follow-up data at the time of the intervention. This study was approved by the institutional review board of the University of Freiburg (ethical approval reference number 69/17, 4 July 2023) and complied with the Declaration of Helsinki.

### 2.1. Pre- and Post-TAVI-CTA

CTA was performed with a dual-source CT scanner (Somatom Definition Flash until September 2020 and Somatom Force thereafter; Siemens Healthineers, Forchheim, Germany). The protocol and the fusion imaging method for pre- and post-TAVI CTA have been described previously [4,6]. In brief, a contrast-enhanced, retrospective ECG-gated CTA that covered the aortic root was performed. The beginning of the scan was defined by bolus tracking within the left atrium. The examination was conducted with a total of 50 mL iodinated contrast agent (Imeron 400, Bracco, Konstanz, Germany) at a flow rate of 4 mL/s. Reconstruction of the images was conducted in 50 ms steps throughout the cardiac cycle with a stent-specific reconstruction kernel (B46f) for post-TAVI CTA. The slice thickness was 1 mm with an increment of 0.8 mm.

### 2.2. Image Analysis

The CTA images were analyzed in multiplanar reconstruction software using a post-processing workstation (Syngo Multimodality Workplace, Version VB60A, Siemens healthineers, Forchheim, Germany) by two experienced readers blinded to the clinical data (P.B. and P.R., both certified with the highest degree of cardiac CTA of the German Cardiac Society), independently.

In the pre-TAVI images, the aortic annulus (defined by the nadir of the three cusps) was assessed and the amount of calcification in the device landing zone was assessed visually, as previously described [4,7]. In brief, we assessed calcification for each aortic valve cusp region as Grade 0—no calcification, Grade 1—mild calcification, Grade 2—moderate calcification, or Grade 3—severe calcification.

For the fusion imaging method, the post-TAVI images were automatically merged with the pre-TAVI images at the corresponding reconstruction time point in systole. The fused images were then manually adapted to an optimal alignment using landmarks such as coronary arteries and calcification spots [4]. The prosthesis position was assessed by measuring the implantation depth (the distance between the annular plane and the ventricular edge of the THV) of the THV within the fused images. These stent measurements were conducted next to each aortic valve cusp. The optimal stent position of the Sapien 3 THV was defined according to the manufacturer’s recommendation as the mean THV position of 70/30% to 80/20% (aortic/ventricular site) of the measured stent length above/below the aortic annulus. The optimal stent position of the Evolut R THV was defined as a THV position < 4 mm below the aortic annulus, as the results of a previous study by our group showed that this value was the best cut-off for the occurrence of new conduction disturbances after TAVI [8].

### 2.3. Statistical Analysis

Statistical analysis was performed using SPSS software Version 29.0.0.0 (IBM Corp., Armonk, NY, USA) and Stata (StataCorp LCC, College Station, TX, USA, version 18). Categorical variables are expressed as frequencies and percentages, and continuous variables are expressed as the mean ± standard deviation (SD). Normal distribution was tested using the Shapiro–Wilk Test. Comparison between the two groups was achieved using the χ^2^-test (categorical variables), Student’s *t*-test (normally distributed continuous variables), or the Mann–Whitney U test (non-normally distributed continuous variables). For predictors of a high PPM stimulation rate, univariate logistic regression models were conducted. Survival was analyzed and visualized using the Kaplan–Meier method, and a comparison of survival between the groups was achieved using the long-rank test. A *p*-value < 0.05 was considered statistically significant.

## 3. Results

### 3.1. Study Population

A total of 387 patients underwent pre- and post-TAVI CTA suitable for fusion imaging between 2014 and 2022. Among them, 112 patients (29%) underwent implantation of a new PPM pre-discharge after TAVI. PPM data at discharge were available for 107 patients, forming the final study cohort (57.0% female, 81.3 ± 5.6 years). Of these patients, 33 patients (30.8%) had an initial non-high burden PPM stimulation rate (≤20%) before discharge, while 74 patients (69.2%) had a high burden PPM stimulation rate (exceeding 20%). There were no significant differences between these two groups regarding age, sex, prosthesis size or prosthesis type, and left ventricular ejection fraction. A higher degree (III°) atrioventricular block was significantly more often the indication for PPM implantation in the high burden PPM stimulation group (*p* = 0.029) (Table 1).

After 3 months, PPM data were available for 18 patients (54.5%) with an initial non-high pacing burden. Among these patients, 12 (66.7%) maintained a non-high pacing burden, while 6 (33.3%) showed an increase to above 20% pacing burden.

**Figure 1:**
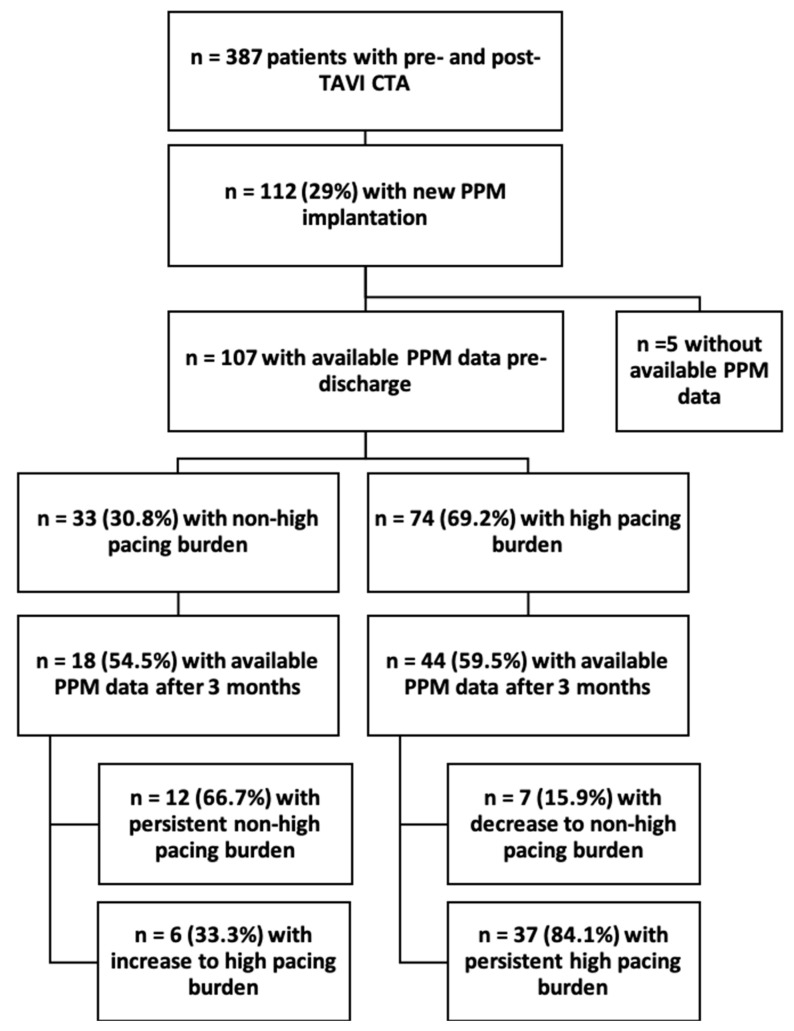
Flowchart of study enrollment.

In the other group with an initial high pacing burden, follow-up pacing data were available for 44 patients (59.5%) after 3 months. Of these patients, 7 patients (15.9%) showed a decrease in the PPM stimulation rate to a non-high pacing burden; in comparison, 37 patients (84.1%) continued to exhibit a high pacing burden. The flow chart of the complete study enrollment is presented in Figure 1.

### 3.2. CTA Measurements

The mean implantation depth of the entire study cohort was 4.3 mm, and the median total degree of calcification was 4.5. Optimal THV position was achieved in 68 (63.6%) of all the patients. There were no significant differences between patients with a non-high pacing burden and those with a high pacing burden pre-discharge (total calcification grade *p* = 0.559; mean implantation depth *p* = 0.550; optimal THV position *p* = 0.990) (Table 2).

At the 3-month follow-up, CTA measurements showed no significant differences between patients with a non-high pacing burden and those with a high pacing burden (total calcification *p* = 0.780; mean implantation depth *p* = 0.901; optimal THV position *p* = 0.934) (Table 3).

### 3.3. Conduction Disturbances

In the entire study cohort, 63 patients (58.9%) had previous conduction disturbances (most common disturbance: I° atrioventricular block (18.7%)). Of note, 90 of these patients (84.1%) developed additional or progressive conduction disturbances after TAVI (most common disturbance: new third-degree atrioventricular block (30.1%)). We did not observe a second-degree atrioventricular block (neither Wenckebach nor Mobitz II) in our patient cohort.

As expected, only a newly developed third-degree atrioventricular block was significantly associated with a high pacing burden pre-discharge (*p* = 0.010) and after 3 months (*p* = 0.035). Our regression analysis results confirmed that this condition was a reliable predictor of a high pacing burden before discharge (*p* = 0.007) and after 3 months (*p* = 0.028) (Table 4 and Table 5).

### 3.4. Follow-Up

Over a mean follow-up period of 3.4 ± 2.2 years, 16 (48.5%) patients in the initial group with a non-high pacing burden and 37 (50.0%) patients in the initial group with a high pacing burden died. Overall survival data were unavailable for 27 patients (25.5% of the entire cohort) and the date of death was unknown in 3 patients (2.8%).

**Figure 2:**
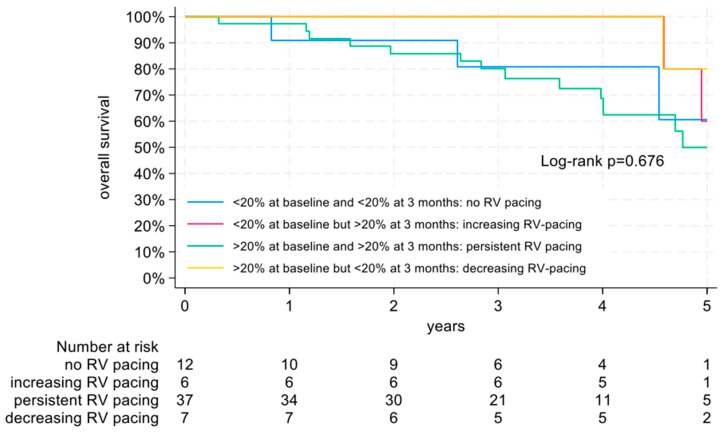
Overall survival depending on the development of PPM (permanent pacemaker) pacing burden.

There were no significant differences in overall survival among the four groups based on the course of PPM pacing burdens within the first 3 months after TAVI (*p* = 0.676) (Figure 2).

## 4. Discussion

To the best of our knowledge, this analysis is the first to examine the impact of three-dimensional THV position, assessed based on fusion imaging from pre- and post-TAVI CTA, on the pacing burdens of newly implanted PPMs after TAVI. The hypothesis-generating key findings of this study are as follows:

1. The implantation depth of the TAVI prosthesis, evaluated based on fusion imaging, did not affect the PPM pacing burden pre-discharge.
2. One-third of the patients with an initial low pacing burden experienced an increase to a >20% pacing burden within three months, suggesting potential long-term conduction system damage.
3. The PPM pacing burden pre-discharge did not impact overall survival in TAVI patients.

Despite numerous advances in optimizing TAVI, the incidence of PPM implantation after TAVI remains approximately 10% [2]. While PPM implantation is generally considered safe, complications such as endocarditis or device dysfunction can occur. These complications are particularly concerning in TAVI patients, who typically have high morbidity and mortality risks. Thus, it is crucial to accurately identify which patients truly require a PPM after TAVI and, when PPM implantation is unavoidable, to identify risk factors associated with PPM complications in these patients.

The pacing rates of PPMs after TAVI vary significantly, leading to several revisions of the recommendations for PPM implantation after TAVI [3]. In this study, pacemaker assessments were included both before discharge and at 3 months, assuming that the initial post-interventional edema would have resolved and any potential THV migration [9] would have stabilized after 3 months. In our analyses, we identified, as expected, a new third-degree atrioventricular block as the sole predictor of a high burden pacing both pre-discharge and after 3 months. This finding is in line with previous studies that have led to a Class IB recommendation in the current guidelines for PPM implantation in patients with a third-degree atrioventricular block persisting for 24–48 h post-TAVI [3]. A new left bundle branch block (LBBB), the most common conduction disturbance after TAVI, showed a trend toward a lower PPM stimulation rate in our data but did not reach statistical significance. This finding is consistent with those of PREVIOUS studies, which suggest that PPM implantation in patients with LBBB alone should be considered only when the QRS duration exceeds 150 ms [3,10,11].

Interestingly, one-third of the patients with an initial low pacing burden (≤20%) experienced an increase to a >20% pacing burden within three months. This finding suggests potential long-term conduction system damage post-TAVI. The results of one previous study showed that shallower implantation of the valve reduced the risk of a high pacing burden (defined as >10%) in patients with pre-existing PPMs [12]. These data emphasize the need for further research regarding the development of pacing burden after TAVI in patients with pre-existing and newly implanted PPMs.

Regarding THV position, the results of previous studies have shown a correlation between a lower implantation depth and the occurrence of new conduction disturbances [8]. The authors of another study also identified an association between implantation depth and PPM pacing burden after TAVI [12]. However, in that particular study, implantation depth was assessed solely using two-dimensional angiography during the TAVI procedure. We determined the THV position in our analysis using fusion imaging of pre- and post-TAVI CTA as this technique provides a three-dimensional visualization of the implanted prosthesis, precisely correlated with the original annulus plane and geometry, serving as the reference for implantation depth [4]. Using the fusion imaging method, we were unable to identify any correlation between prosthesis position and pacing burden.

Generally, PPM implantation is more frequently required after TAVI with self-expandable valves [13]. In our analysis, the prosthesis type (Sapien THV vs. Evolut THV) showed no difference between the PPM stimulation groups. However, the sample size was too small to conduct statistical analyses on the impact of the prosthesis type and size on PPM pacing burden.

The literature on outcomes following PPM implantation post-TAVI is controversial. The authors of a meta-analysis of 17 studies found no increased risk of death associated with PPM implantation in TAVI patients [14]. This finding was confirmed by the results of a multicenter study that demonstrated no increase in mortality but provided evidence of a rise in rehospitalizations following PPM implantation after TAVI [15]. The results of our study align with these findings, showing no differences in survival regardless of the PPM pacing burden or its development over the first 3 months. However, the authors of the aforementioned studies did not include the PPM stimulation rate in their analyses.

In patients with a PPM, a right ventricular pacing burden of >20% is recognized as a predictor for the development of pacemaker-induced cardiomyopathy, which can lead to significant morbidity and mortality [16,17]. The authors of a previous study identified a threshold of >40% PPM pacing burden 6 months after TAVI as being associated with higher rates of hospitalization due to heart failure and cardiovascular death [18]. Conversely, the authors of another study found no increased mortality with a >40% PPM pacing burden after TAVI [19]. There is currently no consensus on a definitive cut-off for harmful right ventricular PPM pacing burden across different patient cohorts. Therefore, we defined a high pacing burden as a >20% PPM stimulation rate, as recommended by the current guidelines of the European Society of Cardiology [3].

In our study, overall survival was unaffected by whether the PPM pacing burden was high or non-high. As a limitation, data at 3 months were only available in a small amount of our patients. Thus, our results should be considered as hypothesis generating. We assume that these conflicting results may be attributed to the age of the patient cohort. In TAVI patients, mortality is often influenced by pre-existing conditions such as coronary artery disease or heart failure, which may affect outcomes. However, as TAVI indications extend to younger patients, this issue could become more relevant in the future, thus warranting further investigation into younger patient cohorts.

### 4.1. Impact on Daily Practice

THV implantations at the recommended depths are sufficient and do not require adjustments to prevent complications such as PPM cardiomyopathy. The implantation depth affects the risk of new PPM implantation, as shown previously [8]; however, once implanted, it does not influence the PPM pacing burden, as shown herein.

PPM implantation can be promptly performed in patients who develop a new third-degree atrioventricular block after TAVI, as only these patients show a correlation with a persistent high-pacing burden. This finding indicates that, in these cases, the influence of post-interventional factors, such as the resorption of post-interventional edema, is negligible.

Since pacing burden does not impact overall survival in TAVI patients, the risks and negative effects of PPM implantation in this older and more comorbid population appear to be less pronounced. Regular cardiac follow-up checks are sufficient for such patients.

### 4.2. Limitations

Several limitations of the present study must be considered. First, we report on a retrospective study, and the findings require external validation. The relatively small size of the study cohort limits the statistical power and prevents analyses with adjustments for baseline variables. Thus, we cannot rule out the presence of minor associations. The small size of the study cohort is based on the inclusion criteria, as only patients with both post-TAVI CTA and PPM were analyzed. Previously published exclusion criteria for post-TAVI-CT [6] further limited the cohort size. The presented rates of pacemaker implantation are higher compared to the literature. This discrepancy may partially reflect changes in clinical practice during the study period (2014–2022), as the initial guidelines recommended permanent pacemaker implantation for all patients with a new left bundle branch block, a practice that was later abandoned as the guidelines evolved.

As PPM controls after 3 months were not conducted at our institution in all patients, we contacted general practitioners to receive these data. However, as our study includes patients treated between 2014 and 2022, we were not able to receive all of these data, which further limits the statistical power of the follow-up after 3 months, as well as the analyses on survival (data unavailable in 25.5% of patients). Additionally, the low incidence of myocardial infarction, bleeding, and stroke during follow-up prevented us from performing statistical analyses on these outcomes in relation to the PPM pacing burden. We observed a high rate of PPM implantation post-TAVI as the indications for PPM implantation have changed over the years, as depicted in this study. Due to the retrospective nature of this study, we are not able to reproduce the individual indication for pacing adjustments in the PPM controls. However, adjustments were conducted by an experienced electrophysiologist (>10 years of experience in electrophysiology) and thus, we believe that pacing was adjusted as low as possible to avoid PPM-induced cardiomyopathy. There were also no patients in this study who received a cardiac resynchronization pacemaker.

Despite these limitations, the impact of our study is strengthened by our novel approach of investigating the three-dimensional prosthesis position in relation to pacing burden both pre-discharge and at 3 months.

## 5. Conclusions

The precise three-dimensional THV position, as assessed via CTA fusion imaging, did not affect the mid-term pacing burden in our patients with a new PPM after TAVI. One-third of our patients experienced an increase to a high pacing burden within 3 months, suggesting potential long-term conduction system damage after TAVI and emphasizing the need for further research. While our findings are specific to this cohort, they underscore the need for further research. Importantly, overall survival in this study was unaffected by PPM stimulation rates.

## Figures and Tables

**Table 1:** Baseline and procedural characteristics of the entire study cohort and patients with non-high and high pacing burden pre-discharge.

	All Patients (*n* = 107)	Non-High Pacing Burden (*n* = 33)	High Pacing Burden (*n* = 74)	*p*-Value *
Age (years)	81 ± 5.6	80 ± 6.5	82 ± 5.0	0.671
Female	61 (57.0%)	21 (63.6%)	40 (54.1%)	0.345
BMI (kg/m^2^)	27.5 ± 5.5	28.0 ± 5.3	27.2 ± 5.6	0.723
LVEF (%)				
Pre-TAVI	51.5 ± 7.3	51.5 ± 7.0	51.5 ± 7.5	0.867
Post-TAVI	52.2 ± 6.1	52.2 ± 6.1	52.3 ± 6.1	0.904
Prosthesis size				0.137
23 mm	26 (24.3%)	11 (33.3%)	15 (20.3%)	
26 mm	45 (42.0%)	15 (45.5%)	30 (40.5%)	
29 mm	34 (31.8%)	7 (21.2%)	27(36.5%)	
34 mm	2 (1.9%)	0 (0.0%)	2 (2.7%)	
Prosthesis type				0.360
Evolut R	29 (27.1%)	7 (21.2%)	22 (29.7%)	
Sapien S3	78 (72.9%)	26 (78.8%)	52 (67.5%)	
PPM indication				
AVB III	43 (40.2%)	6 (18.2%)	37 (50.0%)	* 0.029
AVB I + LBBB	24 (22.4%)	8 (24.2%)	16 (21.6%)	0.413
LBBB	32 (29.9%)	16 (48.5%)	16 (21.6%)	0.071
Bifascicular block	3 (2.8%)	1 (3.0%)	2 (2.7%)	0.837
RBBB	1 (0.9%)	0 (0.0%)	1 (1.4%)	0.467
TBS	4 (3.7%)	2 (6.1%)	2 (2.7%)	0.831

The values shown are the mean ± standard deviation or frequencies and percentages. * comparison between patients with pacing burden ≤ 20% and >20%. AVB, atrioventricular block; BMI, body mass index; LBBB, left bundle branch block, LVEF, left ventricular ejection fraction; PPM, permanent pacemaker; RBBB, right bundle branch block; TAVI, transcatheter aortic valve implantation; TBS, tachy–brady syndrome.

**Table 2:** Results of computed tomography angiography measurements comparing PPM pacing burden pre-discharge.

	All Patients (*n* = 107)	Non-High Pacing Burden (*n* = 33)	High Pacing Burden (*n* = 74)	*p*-Value *
Grade of calcification of the aortic valve, total	4.5 [4.5;5.1]	4.3 [4.1;5.2]	4.8 [4.5;5.2]	0.559
Left coronary cusp	1.5 [1.4;1.7]	1.3 [1.1;1.6]	1.5 [1.5;1.8]	0.052
Right coronary cusp	1.3 [1.3;1.6]	1.3 [1.1;1.6]	1.5 [1.3;1.6]	0.306
Non-coronary cusp	1.8 [1.7;1.9]	1.8 [1.6;2.1]	1.8 [1.6;1.9]	0.261
Implantation depth below annulus (mm), mean	4.3 [4.0;5.0]	4.7 [3.7;5.6]	4.0 [3.8;5.0]	0.550
Left coronary cusp	4.0 [3.8;4.9]	4.0 [3.6;5.7]	4.0 [3.7;4.9]	0.455
Right coronary cusp	4.0 [4.1;5.2]	5.0 [4.2;6.1]	4.0 [3.7;5.1]	0.108
Non-coronary cusp	4.0 [3.8;4.9]	5.0 [3.2;5.3]	4.0 [3.7;5.1]	0.932
Optimal position	68 (63.6%)	21 (63.6%)	47 (63.5%)	0.990

The values shown are the median ± interquartile range or frequencies and percentages. * comparison between patients with pacing burden ≤ 20% and >20%.

**Table 3:** Results of computed tomography angiography measurements comparing PPM pacing burden after 3 months.

	All Patients (*n* = 62)	Non-High Pacing Burden (*n* = 19)	High Pacing Burden (*n* = 43)	*p*-Value *
Grade of calcification of the aortic valve, total	4.6 [4.4;5.2]	4.5 [4.1;5.6]	4.8 [4.3;5.2]	0.780
Left coronary cusp	1.5 [1.3;1.7]	1.3 [1.1;1.8]	1.5 [1.3;1.7]	0.306
Right coronary cusp	1.3 [1.3;1.6]	1.3 [1.0;1.6]	1.5 [1.3;1.7]	0.169
Non-coronary cusp	1.8 [1.7;2.0]	2.0 [1.7;2.4]	1.8 [1.5;1.9]	0.270
Implantation depth below annulus (mm), mean	5.0 [4.3;5.6]	4.7 [3.7;6.3]	4.7 [4.1;5.8]	0.901
Left coronary cusp	4.4 [4.0;5.5]	4.0 [3.1;6.3]	5.0 [3.9;5.6]	0.575
Right coronary cusp	5.0 [4.5;5.9]	5.0 [4.0;6.7]	5.0 [4.2;6.1]	0.437
Non-coronary cusp	5.0 [4.2;5.7]	5.0 [3.6;6.3]	5.0 [4.0;5.9]	0.554
Optimal position	34 (54.8%)	11 (57.9%)	23 (53.5%)	0.934

The values shown are the median ± interquartile range or frequencies and percentages. * comparison between patients with pacing burden ≤ 20% and >20%.

**Table 4:** Association between pre-discharge PPM pacing burden and conduction disturbances.

	All Patients (*n* = 107)	Non-High Pacing Burden Pre-Discharge (*n* = 33)	High Pacing Burden Pre-Discharge (*n* = 74)	*p*-Value *	*p*-Value Regression Analyses
Previous CD	63 (58.9%)	16 (48.5%)	47 (63.5%)	0.420	
Type of previous CD					
AVB I	20 (18.7%)	5 (15.2%)	15 (20.3%)	0.738	
LBBB	15 (14.0%)	7 (21.2%)	8 (10.8%)	0.130	
AVB + LBBB	13 (12.1%)	3 (9.0%)	10 (13.5%)	0.419	
Bifascicular block	9 (8.4%)	0 (0.0%)	9 (12.2%)	0.065	
RBBB	6 (5.6%)	1 (3.0%)	5 (6.8%)	0.842	
New CD	90 (84.1%)	28 (84.5%)	62 (83.8%)	0.559	
Type of new CD					
AVB I	2 (1.9%)	1 (3.0%)	1 (1.4%)	0.321	
AVB III	33 (30.1%)	3 (9.1%)	30 (40.5%)	* 0.010	* 0.007
LBBB	31 (29.0%)	16 (48.5%)	15 (20.3%)	0.063	
AVB + LBBB	20 (18.7%)	7 (21.2%)	13 (17.6%)	0.741	
Bifascicular block	3 (2.8%)	1 (3.0%)	2 (2.7%)	0.837	
RBBB	1 (0.9%)	0 (0.0%)	1 (1.4%)	0.467	

The values shown are frequencies and percentages. * comparison between patients with pacing burden ≤ 20% and >20%. AVB, atrioventricular block; CD, conduction disturbance; LBBB, left bundle branch block; PPM, permanent pacemaker; RBBB, right bundle branch block.

**Table 5:** Association between PPM pacing burden after 3 months and conduction disturbances.

	All Patients (*n* = 62)	Non-High Pacing Burden After 3 Months (*n* = 19)	High Pacing Burden After 3 Months (*n* = 43)	*p*-Value *	*p*-Value Regression Analyses
Previous CD	40 (64.5%)	11 (57.9%)	29 (67.4%)	0.087	
Type of previous CD					
AVB I	11 (17.7%)	3 (15.8%)	8 (18.6%)	0.682	
LBBB	11 (17.7%)	6 (31.6%)	5 (11.6%)	0.098	
AVB + LBBB	8 (12.9%)	1 (5.3%)	7 (16.3%)	0.108	
Bifascicular block	6 (9.7%)	0 (0.0%)	6 (14.0%)	0.085	
RBBB	4 (6.5%)	1 (5.3%)	3 (7.0%)	0.743	
New CD	57 (91.9%)	19 (100.0%)	38 (88.4%)	0.639	
Type of new CD					
AVB I	3 (4.8%)	2 (10.5%)	1 (2.3%)	0.184	
AVB III	18 (29.0%)	1 (5.3%)	17 (39.5%)	* 0.035	* 0.028
LBBB	17 (27.4%)	9 (47.4%)	8 (18.6%)	0.156	
AVB + LBBB	16 (25.8%)	7 (36.8%)	9 (20.9%)	0.137	
Bifascicular block	2 (3.2%)	0 (0.0%)	2 (4.7%)	0.201	
RBBB	1 (1.6%)	0 (0.0%)	1 (2.3%)	0.403	

The values shown are frequencies and percentages * comparison between patients with pacing burden ≤ 20% and >20%. AVB, atrioventricular block; CD, conduction disturbance; LBBB, left bundle branch block; PPM, permanent pacemaker; RBBB, right bundle branch block.

## Data Availability

The data are not publicly available due to them containing information that could compromise the privacy of the research participants.

## Abbreviations

AVB	atrioventricular block
BMI	body mass index
CD	conduction disturbance
CTA	computed tomography angiography
LBBB	left bundle branch block
LVEF	left ventricular ejection fraction
PPM	permanent pacemaker
RBBB	right bundle branch block
RV	right ventricular
TAVI	transcatheter aortic valve implantation
THV	transcatheter heart valve
